# Cytomegalovirus infection in patients with malignant lymphomas who have not received hematopoietic stem cell transplantation

**DOI:** 10.1186/s12885-022-10008-5

**Published:** 2022-09-01

**Authors:** Kazuya Sato, Sho Igarashi, Nodoka Tsukada, Junki Inamura, Masayo Yamamoto, Motohiro Shindo, Kentaro Moriichi, Yusuke Mizukami, Mikihiro Fujiya, Yoshihiro Torimoto

**Affiliations:** 1 Department of Hematology/Oncology, Asahikawa Kosei Hospital, 1-24, Asahikawa, 078-8211 Japan; 2 Division of Metabolism and Biosystemic Science, Gastroenterology and Hematology/Oncology, Department of Medicine, Asahikawa Medical University, Asahikawa, Japan

**Keywords:** Malignant lymphoma, Cytomegalovirus infection, Steroid pretreatment, Multiple therapeutic regimens

## Abstract

**Background:**

Life-threatening cytomegalovirus infection (CMVI) has been reported even in patients with malignant lymphoma (ML) who have not received hematopoietic stem cell transplantation (w/o HSCT) but had been treated with chemotherapy or radiotherapy. However, the CMVI incidence and risk factors (RFs) in patients with ML w/o HSCT have not been fully elucidated. This study aimed to evaluate the clinical aspects, including incidence and RFs, of CMVI in patients with ML w/o HSCT.

**Methods:**

We retrospectively reviewed all patients with ML who received chemotherapy or radiotherapy in our department from 2005 to 2013. The overall survival (OS), incidence and RFs of CMVI, and other characteristics of patients with CMVI were analyzed.

**Results:**

Overall, 236 patients with ML w/o HSCT were evaluated. Of these, 5.5% (13/236) developed CMVI; 54% (7/13) received steroid pretreatment before primary therapy (PT) for ML; and 62% (8/13) received > 2 therapeutic regimens for ML. The OS curve of patients with CMVI was significantly worse than that of patients without CMVI (*p* < 0.0001, log-rank test). A univariate analysis identified B symptoms (*p* = 0.00321), serum albumin < 3.5 g/dL (*p* = 0.0007837), C-reactive protein level > the upper limit of normal (*p* = 0.0006962), steroid pretreatment before PT for ML (*p* = 0.0004262), > 2 therapeutic regimens for ML (*p* = 0.0000818), T cell lymphoma (*p* = 0.006406), and non-complete remission (*p* = 0.02311) as RFs for CMVI. A multivariate analysis identified steroid pretreatment before PT for ML [odds ratio (OR): 4.71 (95% confidence interval [CI]: 1.06–21.0); *p* = 0.0419] and > 2 therapeutic regimens for ML [OR: 9.25 (95% CI: 2.33–36.8); *p* = 0.00159] as independent RFs for CMVI in patients with ML w/o HSCT.

**Conclusions:**

Attention should be paid to CMVI development in patients with ML w/o HSCT pretreated with steroids or who had multiple therapeutic regimens.

## Background

The widespread use of immunosuppressive treatments, such as rituximab, for elderly patients with malignant lymphoma (ML) has led to recent reports of life-threatening cytomegalovirus infection (CMVI) even in patients with ML who had not received hematopoietic stem cell transplantation (HSCT) [1–3]. Factors associated with HSCT, such as transplantation from a CMV-seropositive donor, recipient CMV seropositivity, presence of chronic graft-versus-host disease, use of alemtuzumab or high-dose corticosteroids, or duration of neutropenia, are known risk factors (RFs) for the development of CMVI [4–6].

Several retrospective studies of CMVI in populations that included patients with ML who have received HSCT have reported the incidence rates of CMVI ranging from 3.9 to 16% [6–8]. Tay et al. reported that the incidence rate of CMVI in patients with ML who have received chemoimmunotherapy, but not HSCT, was 9.0% [9]. However, the clinical aspects, including CMVI incidence, survival, and RFs for CMVI, have not been fully elucidated in patients with ML who have not received HSCT but who had been treated with chemotherapy or radiotherapy. It is essential to clarify the clinical aspects of CMVI development in this population to ensure safe administration of chemotherapies and radiotherapies. Therefore, we performed a retrospective clinical analysis to evaluate these clinical aspects, including RFs, of CMVI in patients with ML who had not received HSCT but who had been treated with chemotherapy or radiotherapy.

## Methods

### Patients

We reviewed data of all patients with ML who received chemotherapy or radiotherapy in our department from April 2005 to March 2013 and identified 236 patients (B cell ML:T/NK cell ML:Hodgkin lymphoma [HL] ratio = 195:25:16) who had not received autologous or allogeneic HSCT. Diagnoses of lymphomas were made according to the 4^th^ edition of the World Health Organization Classification of Tumours of Haematopoietic and Lymphoid Tissues [10]. The clinical backgrounds, incidence of CMVI, treatments, overall survival (OS), RFs for CMVI, causes of death, and other characteristics of patients with or without CMVI were analyzed. This study was approved by our institutional ethics review board and was conducted in accordance with the Declaration of Helsinki. The requirement for obtaining patient informed consent was waived due to the retrospective design of the data collection.

### Treatment and outcome

Patients with ML were treated with conventional chemotherapeutic regimens according to the physicians’ discretion; these included R-CHOP (rituximab, cyclophosphamide, doxorubicin, vincristine, and prednisolone) for B cell MLs, CHOP for T cell lymphomas, and ABVD (doxorubicin, bleomycin, vinblastine, and dacarbazine) for HLs. Steroid pretreatment means a steroid treatment temporarily used before the chemotherapy to maintain the patients’ condition. OS was calculated from the first date of diagnosis until death from any cause, or the date of the last contact for surviving patients. The last date of censoring for patients who did not die, but who developed a censoring event, was May 4, 2013.

### Data collection

Clinical or laboratory data, histological findings, treatment, and outcome of every patient were extracted from the electronic medical record system and medical charts at our institute. Histological findings were based on pathology reports. CMVI was defined as virus isolation or detection of viral proteins (antigens) or nucleic acid in any body fluid or tissue specimen [11–13]. Screening for CMV antigenemia was not done routinely but mainly performed in patients with clinical symptoms and signs, such as fever, abdominal pain, diarrhea, liver dysfunction, bone marrow suppression, or interstitial pneumonia, which cannot be fully explained by other pathogens; this screening procedure was also done during the observation period of CMVI. CMV antigenemia was determined by measuring CMV antigen-positive cells per 5 × 10^4^ leukocytes with an anti-pp65 peroxidase-conjugated monoclonal antibody C7-HRP (Teijin antigenemia kit; Teijin, Osaka, Japan) [14]. This study did not assess the CMV serostatus because serum anti-CMV antibodies were not routinely tested in our department. To review cases of CMVI in patients with ML who have not received HSCT, we searched related literatures in PubMed by using the keywords “cytomegalovirus disease,” “cytomegalovirus infection,” and “lymphoma.” Case reports were excluded. Then, we checked and extracted papers reporting on relatively large-scale studies that described the incidence of CMVI, RFs for CMVI, and presence or absence of HSCT for lymphoma. Based on the extracted papers, we summarized the number of patients, incidence of CMVI, RFs for CMVI, and disadvantages in survival prognosis in patients with ML with CMVI in Table 4.

### Statistical analysis

OS was estimated using the Kaplan–Meier method [15], and time-to-event distributions were compared with the log-rank test [16]. RFs for CMVI were initially identified through a univariate analysis. The Fisher’s exact test and t-test were used for categorical and continuous data, respectively; these identified RFs were subsequently evaluated in a multivariate Cox proportional hazards regression analysis using stepwise selection. A t-test was used to compare continuous variables. We divided all parameters into two groups so that it is easy to use the indicators for clinicians. Therefore, all comparisons were performed between two groups, and multiple comparisons were not used. A *p*-value of < 0.05 was considered to indicate statistical significance in all analyses. All statistical analyses were performed using the EZR software package (Saitama Medical Center, Jichi Medical University) [17].

## Results

### Patient characteristics

The clinical background information of all patients with ML is shown in Table 1. The median age of the study participants was 69 years, and 53% (126/236) of the patients were men. Most patients had a good Eastern Cooperative Oncology Group performance status (73%; 173/236), B symptoms (30%; 71/236), and advanced-stage disease (62%; 146/236). B cell lymphomas (83%; 195/236), including diffuse large B cell lymphomas (*n* = 128) and follicular lymphomas (*n* = 47), comprised the largest subcategory of MLs, followed by T/NK cell lymphomas (11%; 25/236) and HLs (7%; 16/236). Most patients were treated with rituximab (76%; 180/236); this included R-CHOP (like) regimens (69%; 163/236). Moreover, 9% (21/236) of the patients received treatment with bendamustine or fludarabine. In addition, 14% (32/236) of the patients received steroid pretreatment before primary therapy (PT) for ML. Radiation therapy was administered to 39% (92/236) of the patients, who had received a median of 1 therapeutic regimen (range: 0–7). Furthermore, 23% (55/236) of the patients received a dose reduction in PT for ML.

**Table 1:**
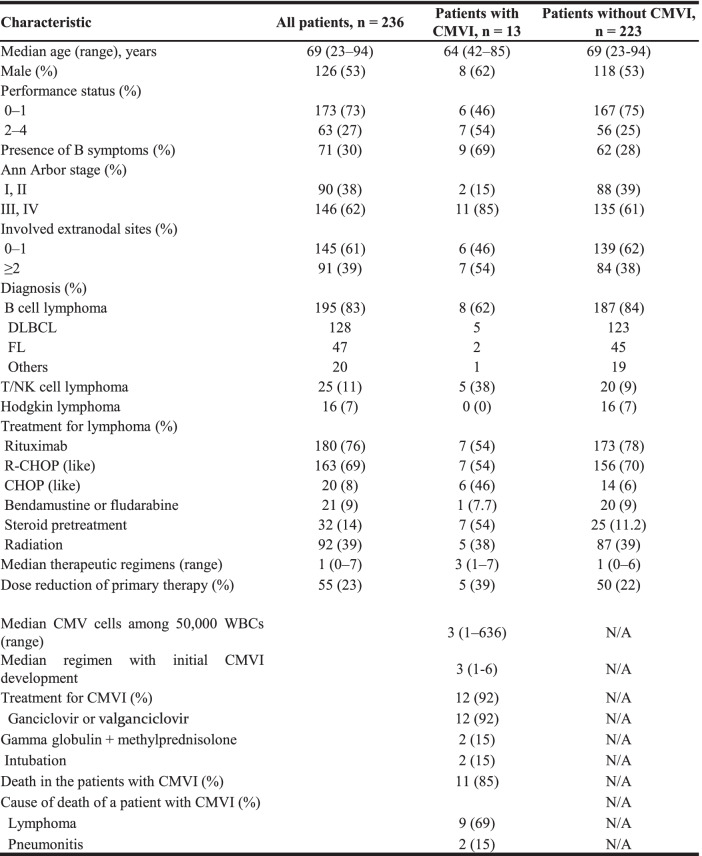
Clinical background of patients with ML

*Abbreviations*: *ML* Maligant lymphoma, *DLBCL* Diffuse large B**-**cell lymphoma, *FL* Follicular lymphoma, *R-CHOP* Rituximab, cyclophosphamide, doxorubicin, vincristine and prednisolone, *CMVI* Cytomegalovirus infection, *WBC* White blood cells

### Characteristics of patients with CMVI

The characteristics of patients with CMVI are shown in Table 1. CMVI developed in 5.5% (13/236) of patients with ML; this group included 8 (62%) men, featured 8 (62%) cases of B cell ML and 5 (38%) of T cell ML, and had a median age of 64 (range: 42–85) years. Approximately half (54%; 7/13) of these patients had received steroid pretreatment before PT for ML, and 62% (8/13) had received > 2 therapeutic regimens for ML. Twelve (92%) patients had received anti-CMV treatments. The median number of CMV antigen-positive cells per 5 × 10^4^ leukocytes at the initial development of CMVI was 3 (range: 1–636). Most patients (92%; 12/13) with CMVI received ganciclovir or valganciclovir, and 2 (15%) were intubated because of pneumonitis. The majority (85%; 11/13) of patients with CMVI eventually died from lymphoma progression (*n* = 9) or pneumonitis (*n* = 2). The putative affected organs by CMV were the liver (hepatitis) in seven and the lung (pneumonitis) in five, whereas four patients had fever only. The median duration of treatment for CMV was 15.5 (range: 0–35) days. Regarding the response to treatment for CMV, 7 out of 13 patients recovered, but the rest did not recover from CMVI. Regarding the treatment of lymphoma after CMVI, of the five patients who developed CMVI during the first-line treatment, two received no treatment, and each of the remaining three patients received CHASE, consisting of cyclophosphamide, cytosine arabinoside, etoposide, and dexamethasone, ESHAP, consisting of etoposide, methylprednisolone, cytarabine, and cisplatin, or oral etoposide. Both patients who developed CMVI during the second-line treatment received oral etoposide, but one died without recovering from CMVI. Of the six patients who developed CMVI after the third-line treatment, four patients died without recovering from CMVI during the final treatment regimen for lymphoma (methylprednisolone, rituximab, CHASE, or EPOCH, consisting of etoposide, prednisone, vincristine, cyclophosphamide, and doxorubicin, respectively). However, the remaining two patients recovered from CMVI; then, FCM, consisting of fludarabine, cyclophosphamide, and mitoxantrone, or radiotherapy was performed.

In addition, patients with CMVI had a higher C-reactive protein (CRP) level (5.659 vs. 1.913 mg/dL; *p* = 0.0002) and lower hemoglobin (10.862 vs. 12.039 g/dL; *p* = 0.0493) and serum albumin levels (3.1 vs. 3.7 g/dL; *p* = 0.0005) at diagnosis than those without CMVI (Table 2).

**Table 2:**
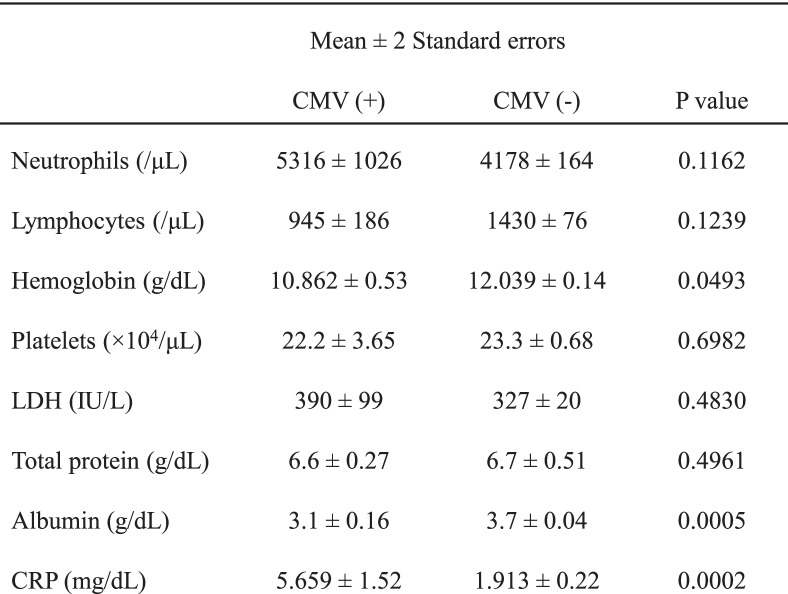
Laboratory data of patients with and without CMV infection at the time of malignant lymphoma diagnosis

*Abbreviations*: *CMV* Cytomegalovirus, *LDH* Lactate dehydrogenase, *CRP C***-**reactive protein

### OS and RFs in patients with CMVI and cumulative incidence of CMVI during follow-up period

**Fig. 1:**
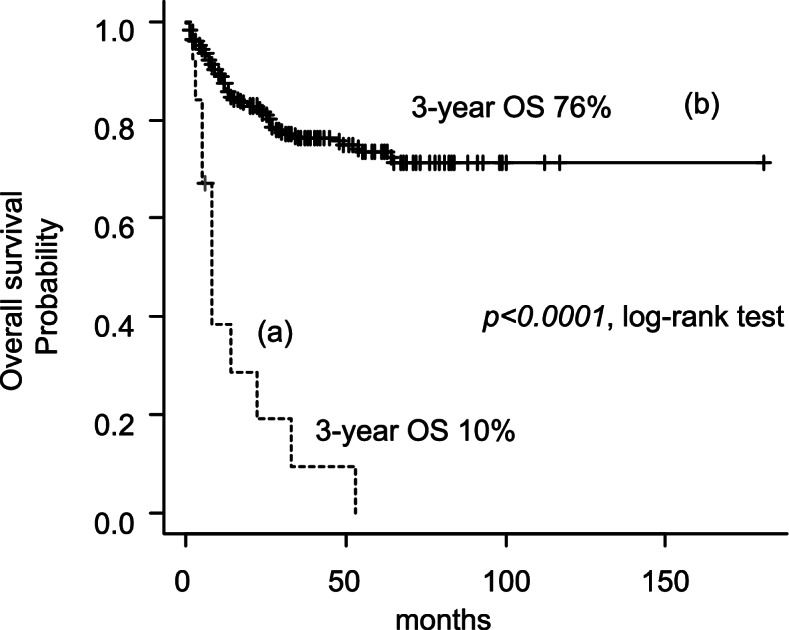
OS of patients with malignant lymphoma, with or without CMV infection. The OS of patients (*n* = 13) with malignant lymphoma who developed CMVI (**a**) was significantly worse (*p* < 0.0001, log-rank test) than that of patients (*n* = 223) without CMVI (b). The estimated 3-year OS rates of patients with and without CMVI were 10 and 76%, respectively. CMVI, cytomegalovirus infection; OS, overall survival

A comparison of OS between patients with and without CMVI revealed significantly worse OS in the former (3-year OS: 10% vs. 76%, *p* < 0.0001 by log-rank test; Fig. 1). To identify the RFs associated with CMVI, the clinical characteristics and background data were compared between patients with and without CMVI in a univariate analysis (Table 3). A significantly higher incidence of CMVI development was observed in patients with B symptoms [odds ratio (OR): 5.79 (95% confidence interval [CI]: 1.55–26.7); *p* = 0.00321)], T cell lymphoma [OR: 6.26 (95% CI: 1.47–24.2); *p* = 0.006406], steroid pretreatment before PT for ML [OR: 9.08 (95% CI: 2.40–35.6); *p* = 0.0004262], > 2 therapeutic regimens for ML [OR: 11.4 (95% CI: 3.04–47.8); *p* = 0.0000818], achievement of first remission [OR: 0.26 (95% CI: 0.07–0.98); *p* = 0.02311], serum albumin level < 3.5 g/dL [OR: 8.02 (95% CI: 1.98–46.8); *p* = 0.0007837], and CRP level of more than the upper limit of normal [OR: infinity (95% CI: 2.57–infinity); *p* = 0.0006962]. The use of rituximab was not a significant RF for CMVI [OR: 0.34 (95% CI: 0.09–1.28); *p* = 0.08545]. These independent seven parameters were subsequently included in a multivariate analysis to determine the independent RFs for CMVI development, which identified steroid pretreatment before PT for ML [OR: 4.71 (95% CI: 1.06–21.0); *p* = 0.0419] and > 2 therapeutic regimens for ML [OR: 9.25 (95% CI: 2.33–36.8); *p* = 0.00159] (Table 3). A cumulative incidence of CMVI during follow-up in the total cohort was 5.5%. In addition, each cumulative incidence of CMVI in patients pretreated with steroid before PT for ML or received > 2 therapeutic regimens for ML was 21.9% (7/32) or 22.9% (8/35), respectively.

**Table 3:**
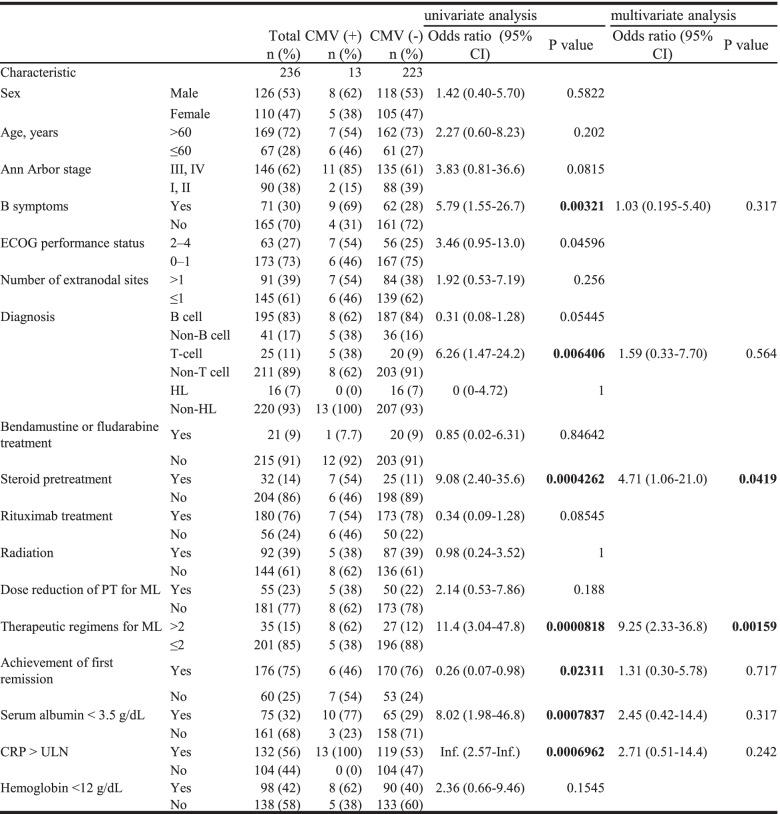
Univariate and multivariate analysis of risk factors for CMV infection

*Abbreviations*: *CMV* Cytomegalovirus, *HL* Hodgkin lymphoma, *PT* Primary therapy, *ML* Malignant lymphoma, *n* Number, *CRP C***-**reactive protein, *ULN* Upper limit of normal, *Inf* Infinity

### CMVI in patients with ML according to the literature

Several investigators had previously reported retrospective studies of CMVI in patients with ML, as shown in Table 4 [6–9]. The reported incidence rate of CMVI ranged from 3.9 to 16% in those studies, which were not limited to patients with ML who had not received HSCT. Although our study included only patients with ML who had not received HSCT, the incidence (5.5%) of CMVI was within the range reported in those earlier studies. In addition, 2 of the 4 previous studies reported RFs of CMVI or CMV disease, such as the use of rituximab-containing regimens [7, 9] or hyper-CVAD (cyclophosphamide, vincristine, adriamycin, and dexamethasone) [9]. However, the authors did not identify the use of steroid pretreatment or number of therapeutic regimens for ML as RFs for CMVI. Two studies have reported the use of rituximab as an RF of CMVI development in patients with ML [7, 9], and CMVI-related fatalities have been reported among patients with ML who were treated with rituximab [1]. Although the use of rituximab-containing regimens was not identified as an RF for CMVI in our study, further investigations are required to clarify whether rituximab use is an RF for CMVI development in patients with ML. In addition, the disadvantages in survival prognosis in patients with ML with CMVI were not obviously reported, except in our study.

**Table 4:**
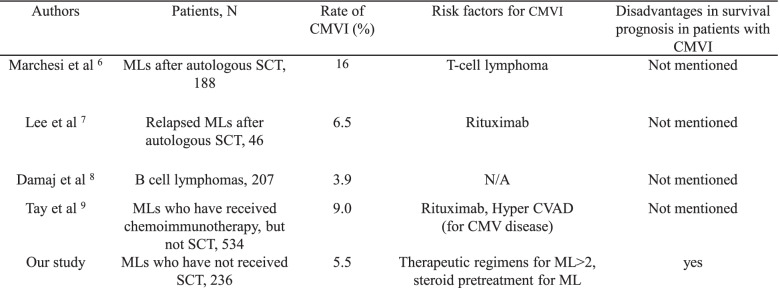
CMVI in patients with ML according to the literature

*Abbreviations*: *CMVI* Cytomegalovirus infection, *ML* Malignant lymphoma, N Number, *SCT* Stem cell transplantation, *N/A* Not available, *CVAD* Cyclophosphamide, vincristine, doxorubicin, and dexamethasone

## Discussion

This retrospective study determined that CMVI occurred in 5.5% (13/236) of the included patients with ML who had received chemotherapy or radiotherapy but not HSCT. In addition, we demonstrated that CMVI development might contribute to poor survival, and that pretreatment with steroids and > 2 therapeutic regimens were independent predictive RFs of CMVI development in these patients. To the best of our knowledge, this is the first report to evaluate the clinical aspects, such as the incidence or RFs, of CMVI in patients with ML who had not received HSCT but who had been treated with chemotherapy or radiotherapy.

We also demonstrated that patients with ML who developed CMVI had a significantly worse OS (*p* < 0.0001, log-rank test) than those who did not develop CMVI, and we noted several potentially related factors. First, patients who developed CMVI had higher rates of advanced-stage disease and poor performance status than those without CMVI (85% vs. 61% and 54% vs. 25%, respectively; Table 3). Second, patients with CMVI had lower levels of hemoglobin and albumin than those without CMVI (10.862 vs. 12.039 g/dL and 3.1 vs. 3.7 g/dL, respectively). These findings might suggest a greater likelihood of CMVI development in patients who already presented with more significant survival disadvantages; accordingly, patients with CMVI exhibited reduced survival. In addition, of the 11 deceased patients, 2 (15%) who had been intubated died of pneumonitis. Therefore, CMVI itself might contribute to reduced survival.

We also indicated that pretreatment with steroids and > 2 therapeutic regimens were independent RFs of CMVI development among patients with ML. In patients with ML with some clinical concerns, such as poor general condition including fever or aggressiveness of lymphoma, steroid pretreatment is used before PT with the expectation of an anti-lymphoma and/or anti-inflammation effect. Therefore, steroid pretreatment and multiple chemotherapeutic regimens might inhibit host immune responses, including CMV-specific T and B cell responses, thus promoting the reactivation of CMV. Given the development of CMVI after steroid pretreatment, standard chemotherapy should be given without steroid pretreatment as much as possible. However, prophylactic treatment for CMVI is not appropriate considering adverse events. Closely monitoring CMV antigen is needed, and antiviral drug treatment should be started at an appropriate timing. The advantages and disadvantages of steroid pretreatment should be confirmed in patients with ML.

Although the duration of steroid treatment was very limited when it was used as a prephase treatment before chemotherapy, steroid pretreatment before PT for ML increases the risk of development of CMVI in this study. We speculate that patients with systemic symptoms, such as fever, or with an aggressiveness of lymphoma may require steroid pretreatment in clinical practice. In these patients, the higher activity of lymphoma rather than the steroid administration itself may lead to immunosuppressive conditions due to progression of lymphoma or an increase in the number of chemotherapeutic regimens. As a result, CMVI might more likely develop.

In the current study, we analyzed patients who were treated for ML between 2005 and 2013. It is interesting to know recent data on the effects of CMV infection in patients with ML who have been treated with drugs with strong immunosuppression such as bendamustine. However, our present study data cannot reveal this. Pezzullo et al. [18] reported an increased incidence of CMV reactivation in older adults (age > 60 years) with non-HL who were treated with bendamustine-containing regimens, especially after the third course of bendamustine accompanied by a significant depression of circulating CD4-positive T cell count and anti-CMV IgG levels. Whether a bendamustine-containing regimen would be an RF for CMVI in our patient cohort needs to be clarified by further analysis in the future.

Several limitations of this study should be acknowledged. First, this was a retrospective study; accordingly, various physician-related selection biases might have existed, such as the frequency or timing of CMV antigenemia testing or treatment strategies for CMVI and ML. Therefore, the incidence of CMVI development and the usefulness of the identified independent RFs for CMVI development should be validated through a prospective analysis of patients with ML who have not received HSCT. Second, CMVI development was not confirmed to be an independent RF for survival among patients with ML who have not received HSCT. Third, the association of the total steroid dose with CMVI development was not confirmed in this study because some relevant data were not available. Fourth, this study had many censored patients with short-term observation in the survival group. It cannot be denied that the risk of CMVI might be underestimated. These issues will require further clarification.

## Conclusion

We retrospectively analyzed patients with ML who had not received HSCT to evaluate the clinical aspects related to CMVI development, including RFs. We demonstrated that the prognosis of patients who developed CMVI was poor and identified two independent RFs for CMVI. Attention should be given to CMVI development in patients with ML who have not received HSCT but who have been pretreated with steroids or multiple therapeutic regimens. Further investigation into the development of CMVI in patients with ML is needed to ensure that chemotherapies and radiotherapies are safely administered.

## Data Availability

The datasets used and/or analyzed during the current study are available from the corresponding author on reasonable request.

## Abbreviations

CMVI: Cytomegalovirus infection
ML: Malignant lymphoma
HSCT: Hematopoietic stem cell transplantation
RFs: Risk factors
OS: Overall survival
PT: Primary therapy
HL: Hodgkin lymphoma
R-CHOP: Rituximab, cyclophosphamide, doxorubicin, vincristine, and prednisolone
ABVD: Doxorubicin, bleomycin, vinblastine, and dacarbazine
CVAD: Cyclophosphamide, vincristine, adriamycin, and dexamethasone

## References

[1] Sharma M, Moore J, Nguyen V, Van Besien K (2009). Fatal CMV pneumonitis in a lymphoma patient treated with rituximab. Am J Hematol.

[2] Le Clech L, Ianotto JC, Quintin-Roue I, Tempescul A (2013). BMJ Case Rep.

[3] Yoshida K, Kosako H, Yamashita Y, Kobata H, Oiwa T, Hosoi H, Murata S, Mushino T, Nishikawa A, Araoka H, Sonoki T, Tamura S (2019). Cytomegalovirus meningoencephalitis in a diffuse large B-cell lymphoma patient undergoing salvage chemotherapy. Rinsho Ketsueki.

[4] Asano-Mori Y, Kanda Y, Oshima K, Kako S, Shinohara A, Nakasone H, Sato H, Watanabe T, Hosoya N, Izutsu K, Asai T, Hangaishi A, Motokura T, Chiba S, Kurokawa M (2008). Clinical features of late cytomegalovirus infection after hematopoietic stem cell transplantation. Int J Hematol.

[5] Ljungman P (2014). The role of cytomegalovirus serostatus on outcome of hematopoietic stem cell transplantation. Curr Opin Hematol.

[6] Marchesi F, Pimpinelli F, Di Domenico EG, Renzi D, Gallo MT, Giulia Regazzo G, Rizzo MG, Gumenyuk S, Toma L, Marino M, Cordone I, Cantonetti M, Liberati AM, Montanaro M, Ceribelli A, Prignano G, Palombi F, Romano A, Papa E, Pisani F, Spadea A, Arcese W, Ensoli F, Mengarelli A (2019). Association between CMV and invasive fungal infections after autologous stem cell transplant in lymphoproliferative malignancies: opportunistic partnership or cause-effect relationship?. Int J Mol Sci.

[7] Lee MY, Chiou TJ, Hsiao LT, Yang MH, Lin PC, Poh SB, Yen CC, Liu JH, Teng HW, Chao TC, Wang WS, Chen PM (2008). Rituximab therapy increased post-transplant cytomegalovirus complications in Non-Hodgkin's lymphoma patients receiving autologous hematopoietic stem cell transplantation. Ann Hematol.

[8] Damaj G, Charbonnier A, Bouabdallah R, Vey N, Coso D, Stoppa AM, Gastaut JA (2004). Monoclonal antibodies and cytomegalovirus infections. Eur J Haematol.

[9] Tay MR, Lim ST, Tao M, Quek RH, Tay K, Tan TT (2014). Cytomegalovirus infection and end-organ disease in Asian patients with lymphoma receiving chemotherapy. Leuk Lymphoma.

[10] Swerdlow SH, Campo E, Harris NL, Jaffe ES, Pileri SA, Stein H, Thiele J, Vardiman JW (2008). WHO Classification of Tumours of Haematopoietic and Lymphoid Tissues (ed 4th).

[11] Ljungman P, Boeckh M, Hirsch HH, Josephson F, Lundgren J, Nichols G, Pikis A, Razonable RR, Miller V, Griffiths PD (2017). Disease definitions working group of the cytomegalovirus drug development forum definitions of cytomegalovirus infection and disease in transplant patients for use in clinical trials. Clin Infect Dis.

[12] Ljungman P, Griffiths P, Paya C (2002). Definitions of cytomegalovirus infection and disease in transplant recipients. Infect Dis.

[13] De la Hoz RE, Stephens G, Sherlock C (2002). Diagnosis and treatment approaches of CMV infections in adult patients. J Clin Virol.

[14] Eizuru Y, Minematsu T, Minamishima Y, Ebihara K, Takahashi K, Tamura K, Hosoda K, Masuho Y (1991). Rapid diagnosis of cytomegalovirus infections by direct immunoperoxidase staining with human monoclonal antibody against an immediate-early antigen. Microbiol Immunol.

[15] Kaplan EL, Meier P (1958). Nonparametric estimation from incomplete observations. J Am Stat Assoc.

[16] Cox DR (1972). Regression models and life tables. JR Stat Soc B.

[17] Kanda Y (2013). Investigation of the freely available easy-to-use software 'EZR' for medical statistics. Bone Marrow Transplant.

[18] Pezzullo L, Giudice V, Serio B, Fontana R, Guariglia R, Martorelli MC, Ferrara I, Mettivier L, Bruno A, Bianco R, Vaccaro E, Pagliano P, Montuori N, Filippelli A, Selleri C (2021). Real-world evidence of cytomegalovirus reactivation in non-Hodgkin lymphomas treated with bendamustine-containing regimens. Open Med (Wars).

